# Composites with Natural Fibers and Conventional Materials Applied in a Hard Armor: A Comparison

**DOI:** 10.3390/polym12091920

**Published:** 2020-08-26

**Authors:** Fernanda Santos da Luz, Fabio da Costa Garcia Filho, Michelle Souza Oliveira, Lucio Fabio Cassiano Nascimento, Sergio Neves Monteiro

**Affiliations:** 1 Military Institute of Engineering—IME, Materials Science Program, Praça General Tibúrcio 80, Urca, Rio de Janeiro 22290-270, Brazil; oliveirasmichelle@gmail.com (M.S.O.); lucio_coppe@yahoo.com.br (L.F.C.N.); snevesmonteiro@gmail.com (S.N.M.); 2 Department of Mechanical and Aerospace Engineering, University of California San Diego—UCSD, La Jolla, CA 92093-0411, USA; fdacostagarciafilho@eng.ucsd.edu

**Keywords:** natural fiber composite, ballistic armor, high-impact ammunition, PALF, UHMWPE

## Abstract

Natural-fiber-reinforced polymer composites have recently drawn attention as new materials for ballistic armor due to sustainability benefits and lower cost as compared to conventional synthetic fibers, such as aramid and ultra-high-molecular-weight polyethylene (UHMWPE). In the present work, a comparison was carried out between the ballistic performance of UHMWPE composite, commercially known as Dyneema, and epoxy composite reinforced with 30 vol % natural fibers extracted from pineapple leaves (PALF) in a hard armor system. This hard armor system aims to provide additional protection to conventional level IIIA ballistic armor vests, made with Kevlar, by introducing the PALF composite plate, effectively changing the ballistic armor into level III. This level of protection allows the ballistic armor to be safely subjected to higher impact projectiles, such as 7.62 mm caliber rifle ammunition. The results indicate that a hard armor with a ceramic front followed by the PALF/epoxy composite meets the National Institute of Justice (NIJ) international standard for level III protection and performs comparably to that of the Dyneema plate, commonly used in armor vests.

## 1. Introduction 

Since the beginning of human civilization, the scenario of armed conflicts around the world has been marked by the constant evolution of weapons and armor. Nowadays, new materials are being researched and developed in an effort to offer protection against not only high-velocity single projectiles but also multiple bullet strikes, explosive devices, and fragments from explosives [1]. However, the increase in protection capacity of monolithic armor [2,3,4] normally causes an increase in its weight. This is an important matter for the protection of soldiers, for whom mobility is a basic requirement, thus demanding lighter and more flexible materials. Studies have been conducted aiming to develop less dense armor systems by joining different materials to create what is known as a multilayered armor system (MAS), which might offer even more protection [3,4,5].

Since World War II, high-performance composites with low density have been gaining preference in armor vests [3]. Currently, synthetic laminates based on aramid fiber (Kevlar/Twaron) [6,7]; ultra-high-molecular-weight polyethylene (Dyneema/SpectraShield) [8], and PBO fiber (Zylon) [9] are commonly used as backing in bulletproof vests. In a recent review, Benzait and Trabzon [10] stated that the emergence of new materials with outstanding stiffness and strength, as well as light density and high energy absorption, makes them a future choice for ballistic armor materials. They also reported another recent research tendency where natural fiber polymer composites are used as alternatives for replacing Kevlar in multilayered armor systems (MASs). These systems are specially designed with a front ceramic for protection against high-velocity (>800 m/s) ammunitions classified as level III [11], such as 7.62 mm caliber, mostly used by military armed forces. Although much weaker than aramid fiber, the investigated natural fiber polymer composites [12,13,14,15,16,17,18,19,20,21,22,23], as an MAS second layer, display comparable ballistic performance to Kevlar with the same thickness. This performance is measured by the behind armor blunt trauma (indentation) caused in a clay witness, simulating a human body, that was placed behind the MAS target. As required by the standard [11], the indentation, also known as back-face signature (BFS), must be smaller than 44 mm. The reason for the unexpected ballistic performance of natural fiber composites is that the role played by the MAS second layer in impact energy dissipation depends on the fiber’s ability to capture ceramic/bullet fragments, but not on the fiber strength [24]. In addition, the articles cited by Benzait and Trabzon [10] and other recent works [25,26,27,28,29,30,31,32,33] also confirmed the superior ballistic performance of natural fiber composites as an MAS second layer. It is worth mentioning the significant number of publications focused on ballistic performance of hybrid composites reinforced with synthetic and natural fibers [34,35,36,37,38]. A common point of these publications is the marked contribution of the natural lignocellulosic fiber in absorbing the ballistic impact energy. In particular, Naveen et al. [34] indicated that the higher energy absorption of their hybrid composite is attributed to lower cellulose and higher lignin in addition to the randomly interlaced dense weaving of the natural coir fiber (*Cocos nucifera*). Among the strongest natural fibers, the one extracted from the pineapple leaf (*Ananas comosus*), also known as PALF, has been extensively investigated as a reinforcement of polymer composites. Indeed, the PALF can reach an ultimate stress over 1.6 GPa and elastic modulus above 80 GPa [24], which significantly improves the strength and stiffness of any polymer matrix.

Besides, the use of natural lignocellulosic fibers (NLFs) is incentivized by their low cost, which can be up to 70 times cheaper than Dyneema [39], commonly used in armor vests. This and other advantages such as low environmental impact, abundant availability, and low degree of industrialization, make natural-fiber-based composites a promising alternative to replace synthetic fibers [40,41,42,43,44,45]. 

Although the use of natural fiber composites in ballistic armors has been reported in many papers [13,14,15,16,17,18,19,20,21,22,23,24,25,26,27,28,29,30,31,32], most of the published work thus far involves a possible third layer in the MAS. This layer is an aluminum alloy, which compromises the armor vest weight. Considering this fact, the present work reports on the development of a double-layer hard armor system with PALF/epoxy composite plate, which can improve the protection level of conventional Kevlar vests (level IIIA). In fact, this combined armor system is capable of offering protection against 7.62 mm caliber rifle ammunition, without the need to add an Al alloy layer.

## 2. Materials and Methods

**Figure 1:**
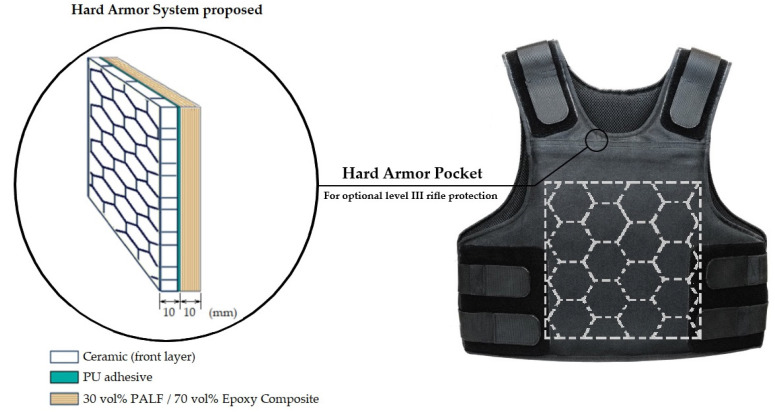
A schematic of the hard armor system proposed and its accommodation in a conventional bulletproof vest (Illustration done by the authors).

The hard armor system (armor plate) consists of two distinct layers, a ceramic front layer followed by a PALF-reinforced epoxy composite, both with the same thickness of 10 mm. These two layers were joined by a thin layer of polyurethane (PU)-based adhesive (Parabrisas Marechal Ltda., Rio de Janeiro, RJ, Brazil). Figure 1 schematically shows the armor plate proposed and where it is accommodated in a conventional level IIIA bulletproof vest to upgrade the protection to level III. As illustrated in Figure 1, the ceramic front layer is composed of an Al_2_O_3_ + 4 wt % Nb_2_O_5_ hexagonal tile mosaic, which was sintered at 1400 °C for 3 h, according to the instructions described in a previous work [46]. The aluminum oxide (Al_2_O_3_) and niobium oxide (Nb_2_O_5_) powders were supplied by the Brazilian firms Treibacher Schleifmittel (São Paulo, SP, Brazil) and CBMM (Poços de Caldas, MG, Brazil), respectively.

**Figure 2:**
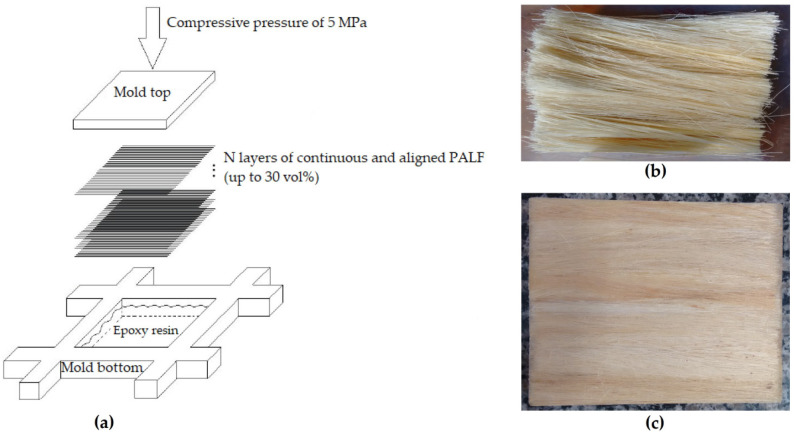
Design and manufacturing of pineapple leaf fiber (PALF) composite: (**a**) hand lay-up process for making composites, (**b**) fibrillated PALF fibers after hand combing, (**c**) 30 vol % PALF/epoxy composite plate (illustration done by the authors).

The composite plates were produced by compression molding using the epoxy resin diglycidyl ether bisphenol-A (DGEBA) with the hardener triethylene tetramine (TETA), added in the stoichiometric ratio phr 13, both supplied by the Epoxyfiber Indústria Química, (Rio de Janeiro, RJ, Brazil). The fiber extracted from pineapple leaves (*Ananas comosus*), also known as PALF, was supplied by Desigan Ltda (Paraná, Brazil), and used as a reinforcement of polymer composites. First, the PALF was cleaned and dried at 60 °C in an air-oven to remove moisture and to not impair interfacial adhesion between the fiber and the matrix, as reported in the literature [47]. In fact, the PALF was not treated; it was used as received aiming for a low degree of industrialization and, consequently, a low production cost. According to previously obtained results by the authors [27], a good interfacial adhesion was attained for this composite even without any fiber treatment. Then, composites with 30 vol % of continuous and aligned fibers and 70 vol % of epoxy were prepared by the hand lay-up process, presented schematically in Figure 2. This process consists of four steps: (1) hand combing of PALF bundle (Figure 2b), (2) manually placing layers of continuous and aligned PALF (up to 30 vol %) into the mold, alternating with the epoxy resin matrix, to guarantee the complete impregnation of the fibers, (3) closing the mold under compressive pressure of 5 MPa, and (4) curing at room temperature for 24 h. 

Ballistic tests were conducted by inserting a PALF composite with a ceramic front layer, as a hard armor plate (Figure 1) in a ballistic vest level IIIA, which was simulated by placing 12 layers of Kevlar S745 fabric, supplied by LFJ Blindagens (Brazil), on the back face of the armor plate. Tests with a single layer of ceramic as a single armor plate were also performed. Additionally, for comparative purposes, a 25 mm thick Dyneema plate was tested. This plate is conventionally used as a hard armor plate in level III vests.

**Figure 3:**
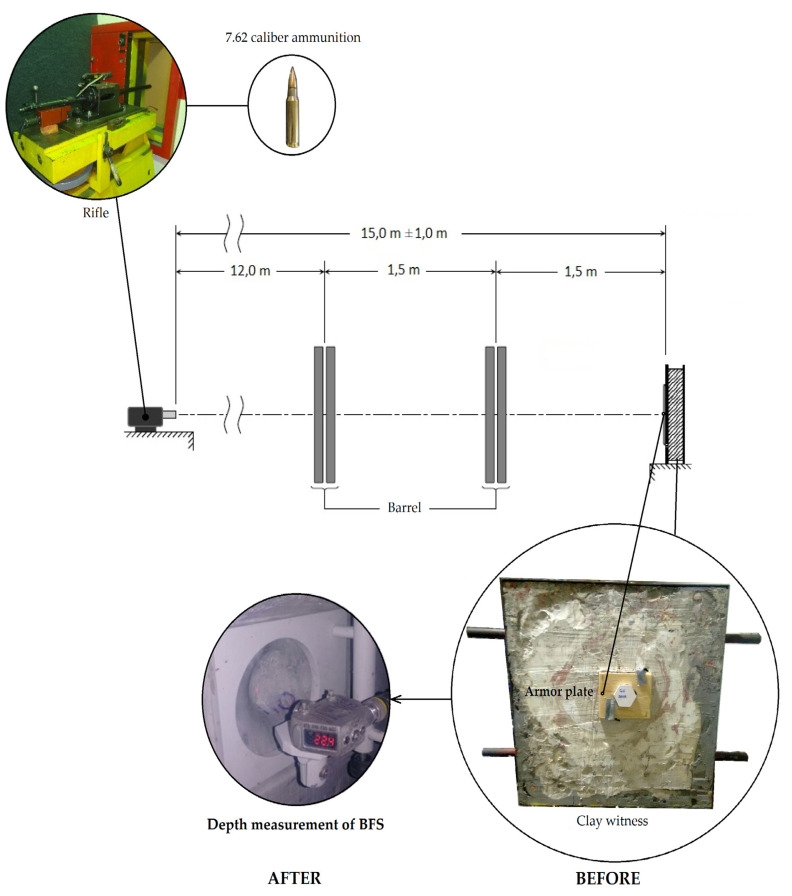
Schematic diagram showing the experimental arrangement (adapted from NIJ standard [11]).

The back-face signature (BFS) test was carried out to measure the armor perforation resistance. In this test, the back face of the armor system was held in direct contact with the clay witness (backing material), which is plastically deformed to capture and measure the depth of trauma (indentation) left after nonperforating ballistic impact [11]. For each test, 7.62 mm caliber ammunition with an impact velocity of 847 ± 9 m/s was used. The armor plate was positioned 15 m from the shooting gun barrel, and the optical barrier system was mounted at 12 m, as shown in the diagram of Figure 3. Ballistic tests were performed at the Brazilian Army Assessment Center (CAEx, Rio de Janeiro, Brazil).

Although the NIJ standard [11] indicated that the group test for level III hard armor should be made up of two armor panels large enough to allow a minimum of six shots, in the present work, tests were performed on reduced armor panels with only one shot per panel. This procedure was done to verify the application feasibility of the PALF-reinforced epoxy composite in a hard armor plate. All other specifications of the NIJ standard [11] were followed. As acceptance criterion, the depth measurement of BFS in the backing material was used. The armor panel was considered efficient when the indentation depth was less than 44 mm, as per standard [11]. 

The depth measurement of indentation was performed by means of a Banner model Q4X laser sensor (Figure 3). In order to verify the difference between results, they were evaluated by statistical analysis of variance (ANOVA).

Both scanning electron microscopy (SEM) and energy dispersive spectroscopy (EDS) were performed in a model Quanta FEG 250, FEI microscope (Field Electron and Ion Co.), Hillsboro, OR, USA.

## 3. Results and Discussion

**Figure 4:**
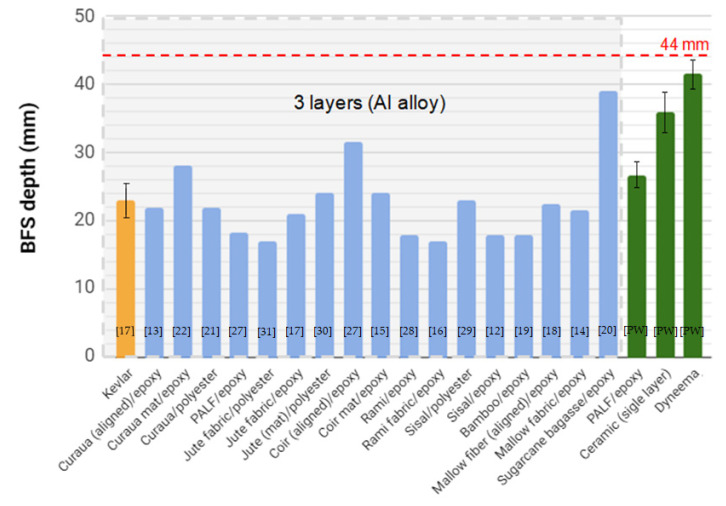
Comparative graph showing multilayered armor systems (MASs) with three layers (ceramic + natural fiber composite + Al alloy) [12,13,14,15,16,17,18,19,20,21,22,27,28,29,30,31] and the armor plates of the present work (PW).

A previous overview [24] showed that a multilayered armor system (MAS) with three layers, consisting of ceramic + NLF composite + Al alloy, already acts as level III protection, meeting the criteria of BFS depth established by NIJ standard [11]. However, level IIIA ballistic vests, which protect against ammunition with an impact velocity lower than 450 m/s, such as 9 mm and .44 Magnum bullets, are often transformed into level III for protection against 7.62 mm bullets by placing inserts (armor plates) on the vest front, which was the objective of the present work. A comparison between the results obtained in previous works for the MASs with three layers [12,13,14,15,16,17,18,19,20,21,22,27,28,29,30,31] and the BFS depth of hard armor systems (without the Al alloy) tested in this work is shown in Figure 4. All MASs presented in this figure have a composite plate with 30 vol % of natural fiber, which is the same as the volume fraction for the tested PALF composite.

For all tested cases, there was no complete perforation of the target. Indeed, the most important result, common in all studies, concerning the ballistic performance of natural fiber composites as an MAS second layer was that they met the standard requirement of indentation depth, i.e., less than 44 mm (Figure 4). It was observed that the majority of MASs with NLF composite exhibited an average BFS depth similar to that of the MAS using Kevlar [17] with the same thickness (10 mm) as the second layer, highlighted in orange in Figure 4. It was also possible to verify that the hard armor plate of Dyneema with 25 mm of thickness met the NIJ criteria, although the average value of 41.5 ± 1.8 mm was on the specified limit [11]. It is worth mentioning that the values obtained for the hard armor system with the proposed PALF composite were significantly different in comparison to the ceramic (single layer) and the Dyneema layer, as shown by the ANOVA results in Table 1, since the *p*-value was lower than 5%. In other words, the ceramic/PALF composite armor plate exhibited higher ballistic performance, with a BFS depth of 26.6 ± 2.0 mm, while the ceramic (single layer) exhibited a value of 35.9 ± 3.0 mm, which indicates the possibility of further reducing the thickness of the ceramic layer in the ceramic/PALF composite armor plate system, consequently reducing its weight.

**Figure 5:**
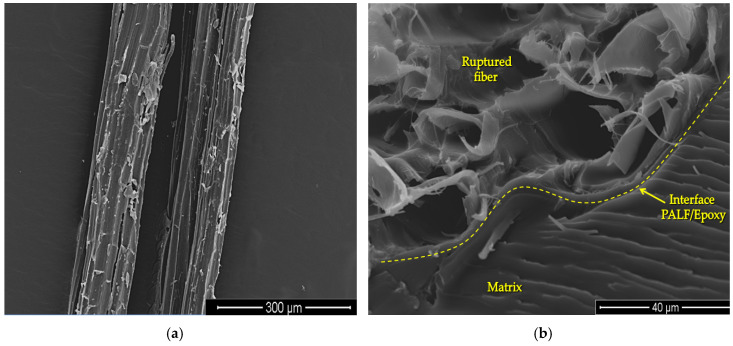
Scanning electron microscopy (SEM) images of PALF morphology: (**a**) rough surface; (**b**) interface adhesion between PALF and epoxy matrix.

**Figure 6:**
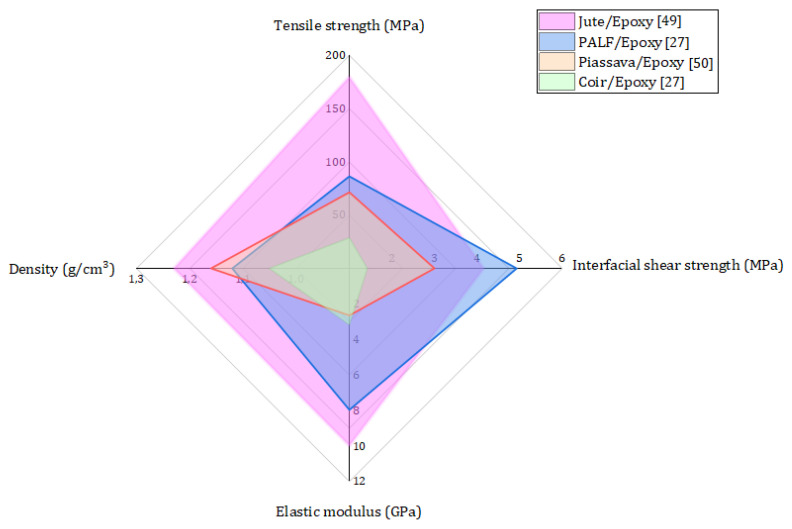
The comparison of physical and mechanical properties between PALF/epoxy [27], jute/epoxy [49], piassava/epoxy [50], and coir/epoxy [27] composites (illustration done by the authors).

The results in Figure 4 show that there is a higher ballistic performance (lower BFS) of the armor plate with PALF composite in comparison to three MAS layers (ceramic/composite/Al alloy) and to sugarcane bagasse/epoxy and coir (aligned/epoxy composites). A possible explanation for this is the strong interfacial interaction between the PALF and the epoxy matrix [27], and the excessively weak adhesion between the epoxy resin and fibers, sugarcane bagasse and coir, which might result in the premature failure of the composite. The interfacial interaction between untreated PALF and epoxy composites was measured by a single fiber pullout test and discussed by the authors elsewhere [27]. In fact, the coir fiber exhibited interfacial shear strength of 1.42 MPa, a value 3.5 times weaker than the PALF, as obtained previously by the authors [27]. The PALF presented a naturally rough surface morphology, as illustrated in Figure 5a, which provides efficient penetration and anchoring of the epoxy matrix, resulting in a good interface adhesion (Figure 5b). This strong adhesion allowed the load to transfer more efficiently from the PALF to the composite matrix, resulting in the PALF composites having a Young’s modulus and tensile strength that were 301% and 251% higher, respectively, than those of the coir composites [27]. A comparison between PALF/epoxy composite and other natural fiber composites is presented in Figure 6. In this comparison, the good relationship is noted between the physical and mechanical properties, such as density, stiffness, and strength, of the PALF composite. It is worth mentioning that, although the jute composite presents higher strength and stiffness, the PALF composite has a lower density and greater interfacial shear strength. In addition, the mechanical testing results are supported by DMA results reported elsewhere [48], which revealed an increase of loss modulus and glass transition temperature values, indicating an efficient load transfer through the PALF/epoxy interface.

Another relevant point regarding the use of natural fiber polymer composites as an MAS second layer in ballistic tests is the impedance of shock waves, which is directly proportional to the material’s density. In fact, due to the relatively lower density of these composites, compared to the ceramic front layer, a compressive shock wave of the projectile impact is reflected as a tensile wave at the interface, helping to fragment the frontal ceramic and increase the amount of energy dissipated [51]. In other words, as these composites have a lower density than Kevlar, their impedance is also lower, which results in a reflected tensile wave with higher amplitude and in the consequent dissipation of more energy. This might be the case for the jute/polyester and ramie/epoxy fabric composites shown in Figure 4.

**Figure 7:**
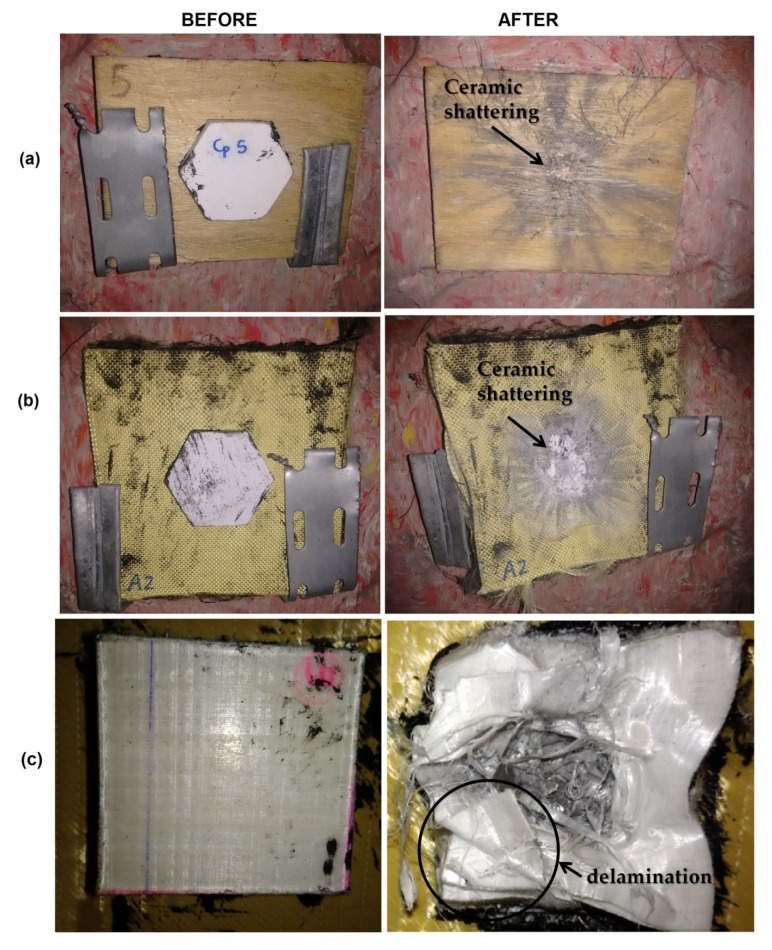
Armor plates before and after the ballistic test. The ceramic/PALF composite system (**a**), A single layer of ceramic (**b**), and a Dyneema plate (**c**).

Figure 7 presents the armor plates before and after the ballistic test for the ceramic/PALF composite system (Figure 7a), a single layer of ceramic (Figure 7b), and a Dyneema plate (Figure 7c). The complete shattering of the ceramic layer is observed after the test (Figure 7a,b). 

**Figure 8:**
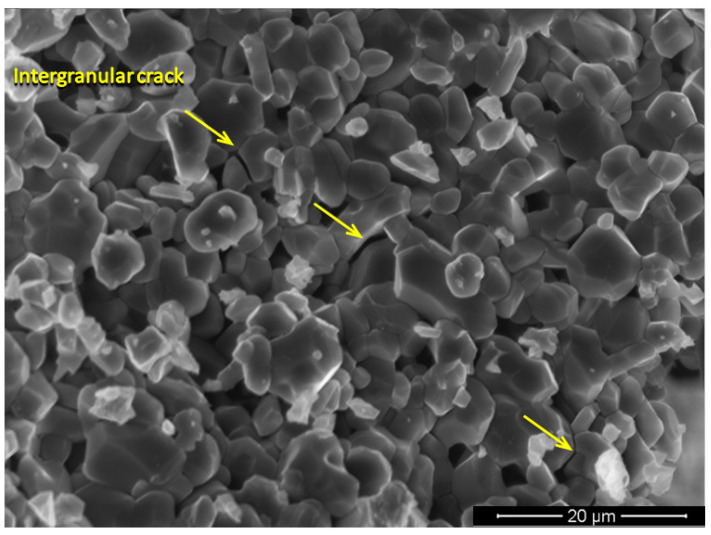
SEM image of ceramic intergranular fragmentation after ballistic test.

The behavior of the ceramic layer is explained by the intergranular fragmentation that occurs due to the Nb_2_O_5_ embrittlement shown in Figure 8. In this fracture mode, it is possible to observe a crack branching throughout the grain boundaries, which helps to dissipate energy and consequently results in better dynamic fracture toughness [47]. 

On the other hand, a depression with a semispherical shape was observed in the frontal face of the Dyneema plate, with the occurrence of delamination between the composite layers (Figure 7c), which is its main energy dissipation mechanism. Although only partial penetration was verified, the blunt trauma (indentation) was, as aforementioned, close to the NIJ standard limit (44 mm) [11], and it was higher than those observed for all composites with natural fibers (Figure 4).

**Figure 9:**
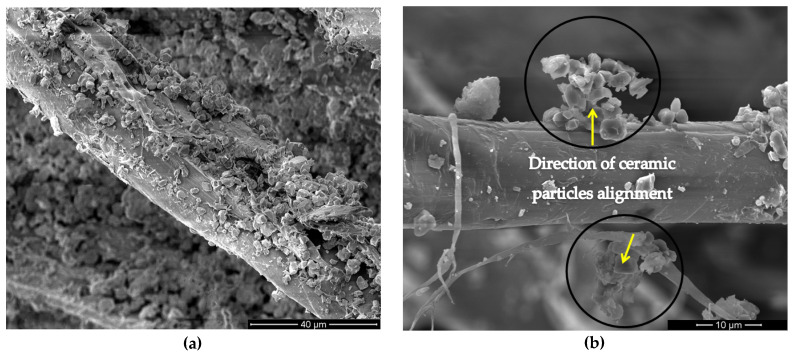
SEM images of the fracture surface of the PALF composite after the ballistic test: (**a**) PALF impregnated with ceramic aggregates; (**b**) alignment of ceramic aggregates on PALF surface.

**Figure 10:**
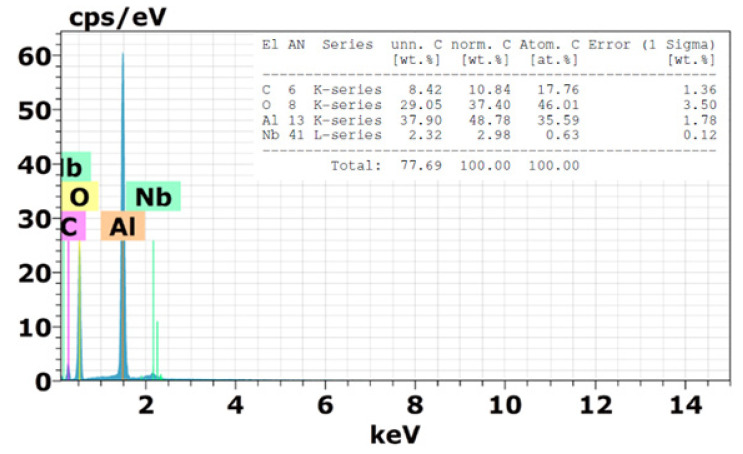
Energy dispersive spectroscopy (EDS) analysis of the PALF surface with ceramic aggregates.

Scanning electron microscopy (SEM) images of fracture surface of the PALF composite after the ballistic test are shown in Figure 9. For this composite, energy absorption mechanisms such as elongation, delamination, pullout, and fiber rupture were identified. However, the main mechanism of energy dissipation was the capture of fragments resulting from the ceramic shattering (Figure 9a), similar to the mechanism disclosed for the Kevlar as the second layer in a MAS [51]. Details of the PALF covered with small ceramic fragments are revealed in Figure 7b. The presence of these ceramic aggregates in the composite surface is assigned to van der Waals force and electrostatic attraction, which contribute to the arrangement and piling of these fragments. Figure 8 presents the energy dispersive spectroscopy (EDS) analysis of these aggregates. The results show that these aggregates must be ceramic (Al_2_O_3_ + Nb_2_O_5_) fragments, given by the content of Al, O, and Nb listed in Figure 10. 

## 4. Conclusions

1. A comparison between the ballistic performance of pineapple leaf fiber (PALF) epoxy composites and ultra-high-molecular-weight polyethylene (Dyneema) backing the front ceramic of multilayered armor systems revealed that no perforation of the target occurred in all armor plates.
2. All the armor plates (single-layer ceramic tested targets, Dyneema, and the ceramic/PALF composite) met the NIJ standard. However, the Dyneema, a material conventionally used in bulletproof vests, tested as a 25 mm thick hard armor plate, exhibited a back-face signature (BFS) depth (41.5 mm) that was close to the NIJ standard limit (44 mm) and performed significantly worse than the ceramic/PALF composite.
3. The hard armor system with the ceramic front layer followed by the epoxy composite incorporated with 30 vol % of pineapple leaf fibers (PALF) exhibited a BFS depth of 26.6 mm, which meets the NIJ standard for ballistic protection against a rifle with 7.62 mm caliber ammunition. Tests using single-layer ceramic as an armor plate presented a 35% higher BFS depth, but the depth was still below the 44 mm required by the standard criterion.
4. Therefore, these results indicate the possibility of optimizing the thickness of this armor plate. They also highlight the potential of using the PALF composite in a hard armor system in order to transform a ballistic vest from level IIIA to level III. However, further experimental work in an enlarged armor plate with six-shot testing is needed to validate the application of this composite in a hard, multilayered armor system.

## Figures and Tables

**Table 1:** Analysis of variance (ANOVA) applied to the back-face signature (BFS) results of all armor plates tested.

Source	Sum of Squares	Degrees of Freedom	Mean of Squares	F (Calculated)	*p*-Value	F Critical
Treatment	799	2	399	74	2 × 10^−9^	3.6
Residual	97	18	5			
Total	896	20				

Note: “Treatment” is the type of hard armor, “total” equals the number of treatments times the number of samples, “residual” is the difference between the “total” and “treatment” values, “degrees of freedom” is the minimum number of independent parameters, “mean of squares” is the ratio of “sum of squares” to “degrees of freedom”, “F calculated” is the ratio between the mean squares of treatments and residues, “F critical” is tabulated (Snedecor F), and “*p*-value” is the probability of obtaining test results at least as extreme as the results actually observed.
